# The RNA-binding protein repertoire of *Arabidopsis thaliana*

**DOI:** 10.1038/srep29766

**Published:** 2016-07-11

**Authors:** Claudius Marondedze, Ludivine Thomas, Natalia L. Serrano, Kathryn S. Lilley, Chris Gehring

**Affiliations:** 1 Cambridge Centre for Proteomics, Cambridge Systems Biology Centre, and Department of Biochemistry, University of Cambridge, Tennis Court Road, Cambridge CB2 1GA, UK; 2 Bioscience Core Laboratory, 4700 King Abdullah University of Science and Technology, 23955-6900 Thuwal, Kingdom of Saudi Arabia; 3 Division of Biological and Environmental Sciences and Engineering, 4700 King Abdullah University of Science and Technology, 23955-6900 Thuwal, Kingdom of Saudi Arabia

## Abstract

RNA-binding proteins (RBPs) have essential roles in determining the fate of RNA from synthesis to decay and have been studied on a protein-by-protein basis, or computationally based on a number of well-characterised RNA-binding domains. Recently, high-throughput methods enabled the capture of mammalian RNA-binding proteomes. To gain insight into the role of *Arabidopsis thaliana* RBPs at the systems level, we have employed interactome capture techniques using cells from different ecotypes grown in cultures and leaves. *In vivo* UV-crosslinking of RNA to RBPs, oligo(dT) capture and mass spectrometry yielded 1,145 different proteins including 550 RBPs that either belong to the functional category ‘RNA-binding’, have known RNA-binding domains or have orthologs identified in mammals, *C. elegans*, or *S. cerevisiae* in addition to 595 novel candidate RBPs. We noted specific subsets of RBPs in cultured cells and leaves and a comparison of Arabidopsis, mammalian, *C. elegans*, and *S. cerevisiae* RBPs reveals a common set of proteins with a role in intermediate metabolism, as well as distinct differences suggesting that RBPs are also species and tissue specific. This study provides a foundation for studies that will advance our understanding of the biological significance of RBPs in plant developmental and stimulus specific responses.

Although transcription is the first and main target of gene expression control, transcripts are also subject to post-transcriptional control including RNA processing, modification and localization. In addition, translational and post-translational regulations as well as the turnover rate of specific proteins add to the complexity of the system. Perhaps surprisingly, previous studies in yeast (*S. cerevisiae*) have shown, that the total amount of transcribed RNA that will eventually be translated is only about 0.5%^1^,^2^. This percentage implies the presence of a tightly regulated post-transcriptional control, that is in parts achieved by RNA-binding proteins (RBPs)^3^,^4^. In eukaryotic systems, RBPs together with noncoding (nc)RNAs such as microRNAs have been reported to direct and regulate the post-transcriptional fate of mRNA in the nucleus and cytoplasm, affecting many processes that include splicing, 3′-end formation, editing, localization and translation (reviewed elswhere^5^). Since RBPs target RBP-binding sites in the untranslated regions (UTRs) of mRNAs that have *cis*-acting regulatory functions, it is likely that the repertoire of the expressed RBPs may be highly specific and informative about developmental and physiological states of cellular systems^6^.

RBPs come in a wide range of combinations of different domains and domain architectures that enable efficient and specific function (for review see^7^) and can bind to single or double stranded (ds)RNA and form dynamic ribonucleoprotein (RNP) complexes^8^. In addition, RBPs contain structural motifs such as RNA recognition motif (RRM), dsRNA-binding-domain and zinc fingers. In animal systems, it has been reported that altering the expression pattern or mutating RBPs or their target binding sites can affect alternative splicing and cause diseases such as e.g. muscular atrophies, neurological disorders or cancers^9^,^10^.

The presence of RBPs in plants has been reported^7^,^11^,^12^,^13^,^14^,^15^,^16^,^17^,^18^, however, the roles and mechanisms of action of these RBPs is considerably less well studied than in animals but plant RBPs may have similarly critical roles in RNA processing including translocation, modification, translation and degradation^19^,^20^,^21^. In the single cell green alga *Chlamydomonas reinhardtii*, RBPs have been shown to regulate chloroplast mRNA stability and translation particularly in response to light^22^. In *Arabidopsis thaliana* alone there are >200 different annotated RBPs of which only a small number have been functionally characterized and most recent studies have focused on the characterization of specific genes encoding RBPs that influence development, hormone-dependent signaling and redox regulation^23^,^24^,^25^ as well as stress responses^26^,^27^,^28^. In addition to their role in normal cellular functions, RBPs are emerging as a class of proteins involved in a wide range of post-transcriptional regulatory events that are important in providing plants with the ability to respond rapidly to changes in environmental conditions^29^. While the role of RBP in plants is increasingly recognized as key to post-transcriptional regulation, to date a systems wide identification of RBPs and inference of their potential role in diverse stimuli responses and cellular processes has not been reported. Recently, technological advances have been reported that allow capturing the RNA-binding proteome. The methods include a combination of UV crosslinking of RNA and RNA interacting proteins, capturing the product using oligo(dT) followed by mass spectrometric identification of the proteins captured. Here we report the RB-proteome and show that UV-crosslinking *in vivo* can be used to identify novel RBPs in cell suspension cultures and in the leaf. We also show the diversity of RNA-binding domains and infer that RBPs have a role in cellular metabolic processes and abiotic stress responses. We also noted some of the Arabidopsis RBPs have dual functions much like some RBPs in animal systems.

## Results and Discussion

### Identification of RNA-binding proteins

In order to identify the RNA-binding proteome of *Arabidopsis thaliana*, we used plant material from cell suspension cultures of Arabidopsis ecotypes Columbia-0 (Col 0) and Landsberg erecta (Ler-0) as well as 4-week old Arabidopsis leaves. We applied the interactome capture technique according to established protocols^30^,^31^. This technique combines UV-crosslinking of RNA to RBP in living cells, oligo(dT) capture and tandem mass spectrometry. This UV-crosslinking protocol has proven effective in selecting proteins that directly bind to RNA while discriminating against proteins that associate as subunits of larger RNA-binding complexes that have no direct contact with RNA molecules. This occurs mainly because the UV-crosslinking protocol does not facilitate protein-protein crosslinking^32^,^33^,^34^. Moreover, the lysis buffer components that include lithium chloride and lithium dodecyl sulphate permit dissociation of non-covalently associating protein-protein interactions^35^. A western blot analysis was performed to assess the sensitivity and selectivity of the UV-crosslinking method applied. As expected, abundance of β-actin and histone 3 proteins were only detected in the total soluble protein extracts and polypyrimidine tract-binding protein 1 was more abundant in the UV-crosslinked samples than in the total soluble protein extracts. This is consistent with an enrichment of RBPs (Supplementary Figure S1A). In addition, the controls, non UV-crosslinked (nUV) samples and RNase T1 and RNase A treated samples, showed no reactivity with the β-actin, histone 3 and polypyrimidine tract-binding protein 1 antibodies. Using tandem mass spectrometry coupled with nanoLC, we identified a total of 1,145 proteins constituting the Arabidopsis RB-proteome. Of these proteins, 705 were detected only in the UV-crosslinked samples and 440 were statistically enriched (p-value ≤ 0.05) upon UV-crosslinking (Supplementary Tables S1 and S2). Of the 1,145 proteins, 401 proteins were identified in the cell suspension culture Col 0, 622 proteins in Ler-0 and 236 proteins in the leaf. Only 18 proteins were common to the three groups (Supplementary Figure S1B, Supplementary Table S1). Thirteen of the common proteins have a role in translation and the ribosomal proteins make the dominant group. In addition, only one protein, pumilio 5 (AT3G20250), containing a classical RBD was detected. Pumilio 5 is a member of the Arabidopsis pumilio proteins containing a PUF domain. PUF proteins regulate both mRNA stability and translation through sequence-specific binding to the 3′UTR of target mRNA transcript. In Arabidopsis, 736 proteins are novel candidate RBPs not previously reported as having a role in RNA processing and they also do not appear to have classical or non-classical RNA-binding domains^8^. Of the novel RBP candidates, 435 proteins were identified in Ler-0, 226 in Col 0 and 114 in the leaf. Perhaps surprisingly, we only report 18 proteins as common in the cell suspensions cultures and the leaf. The number of proteins common in our datasets is rather lower than might be expected since proteins involved in processes like splicing and other conserved RNA metabolic processes might be expected to be common. There are several explanations for this observation. Firstly, the low number of common proteins could, at least partly, be attributed to the low number of proteins identified from the leaf samples and secondly, the stringent parameters employed on assigning proteins - a protein needed to be present in at least two biological replicates to be considered, only one missing cleavage for trypsin was permitted, MOWSE score of ≥32 and a 0.1% FDR. Relaxing some of these parameters, such as considering proteins identified in all replicates and containing one peptide as well as about 10% FDR, will give rise to 80 common proteins despite the low coverage of the leaf samples (Figure S1C). Of the common 80 proteins, 38% (30 proteins) have a functional role in RNA-binding and are mainly ribosomal proteins (constituting 21% of the common proteins). In this study high stringency was applied to obtain a high confidence data set particularly for novel candidates, and this actually excluded some canonical RBP that consequently are not considered in the data reported here.

In these analyses, three biological replicates were tested and a protein was considered present only if it was detected in at least two of the replicates. Further, a stringent statistical criterion was applied to proteins identified in both UV-crosslinked and nUV samples in order to differentiate *bona fide* RBPs from non-specifically bound proteins. We applied a fold increase of >1.5 as threshold and a statistical confidence of p ≤ 0.05 for proteins that were detected in both UV and nUV data sets to classify proteins as UV enriched. These criteria largely exclude false positives but cannot exclude false negatives. Examples of such false negatives include RNA-binding plectin (AT4G25740) that contains the Plectin/S10, N-terminal domain that has been proposed to bind RNA as part of its role in the ribosomal subunit assembly and translation^36^ and the RNA-binding family protein (AT4G17720) that contains classical RNA recognition motif (RRM) domains and is implicated in the response to cytokinin^37^. Nevertheless, we cannot rule out that these false negatives are indeed false, as they may interact with RNA under different physiological conditions. As previously suggested^35^, this approach has some limitations that include failure to identify RBPs when their target RNA molecule is not expressed or the RBP fails to be crosslinked at 254 nm wavelength^35^.

### The domain organization of RBPs

**Figure 1:**
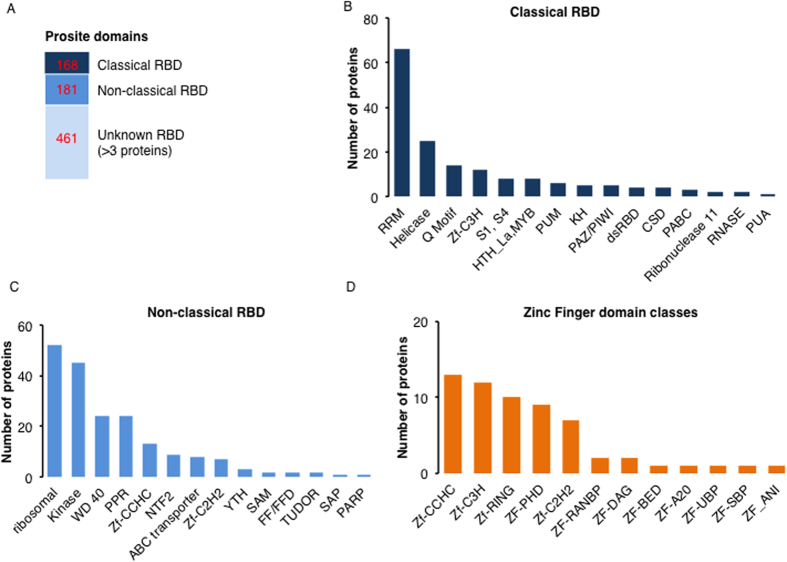
Globular domains in *Arabidopsis thaliana* Col 0 and Ler-0 mRNA interactome proteins. (**A**) Number of proteins containing classical, non-classical, or domains unknown to be RBDs in the mRNA interactome. This figure was constructed based on previous classifications^35^. (**B**) Number of proteins harboring classical RBD in the mRNA interactome. (**C**) Number of proteins harboring non-classical RBD. Some proteins here do contain classical RBD in addition to the non-classical RBD. (**D**) Number of proteins harboring of Znf motifs as well as the distribution of its occurrence within the Arabidopsis mRNA interactome. Only prosite domains with at least three hits were considered.

**Figure 2:**
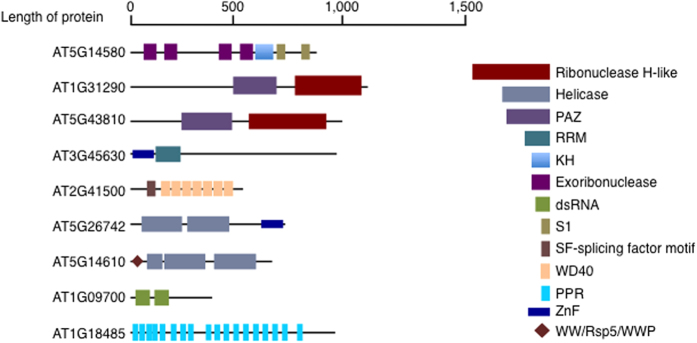
Domain organization in selected mRNA interactome proteins. The number of RBD vary from one, which could be just a single domain or repeated several times such as in AT1G18485, to diverse domains per protein for example three domains as in AT5G14580. The key on the right (not drawn to proportion of the domain length) represent some of the different domains that proteins harbor and the bar represent an approximate length of each protein.

A search for classical, non-classical and unknown RBP domains in the RB-proteome was performed using prosite (http://prosite.expasy.org). The classification of RNA-binding domains (RBDs) was carried out as detailed previously^8^,^35^. In this study, >350 of the RB-proteome proteins harbor known RBDs, including the proteins with classical RBDs such as the RRM, helicases and pumilio (PUM) and non-classical RBDs like ribosomal domains, kinase domains and tryptophan-aspartic acid 40 (WD40) domains (Fig. 1A–C). The helicases, enzymes that catalyze the cleavage of double-stranded nucleic acids in an energy-dependent manner, identified here include the DEAD and DEAH box containing families, superfamily (SF) 1 and 2 helicase harboring the ATP-binding type-1 domain profile as well as the C-terminal domain profile^38^. The SF1 and 2 superfamilies constitute the largest group of DNA and RNA helicases in all species^39^. All helicases bind ATP and harbor a phosphate-binding loop or P-loop (referred to as the classical Walker A) and Mg^2+^-binding aspartate motifs (Walker B)^39^. Diverse classes of the classical RBDs, including zinc fingers, were also detected (Fig. 1D) in addition to, unknown RBDs (Supplementary Table S3). The unknown RBD classes contain mainly proteases (zinc, Clp and thiol proteases), glycosyl hydrolases, translational (tr)-type guanine nucleotide-binding (G) domain (G_TR_2) and amino acyl tRNA ligases that are generally not known to interact with mRNA molecules (Supplementary Table S3). In Arabidopsis, a search for the term RRM returns 256 candidate RBPs, representing the most prevalent classical RBD and similarly for rice (*Oryza sativa*) with 322 proteins. It is also noteworthy that multiple RBDs were detected in some RBPs identified in this study, while other proteins contain a single domain repeated multiple times much like previously reported^18^,^35^. Examples of these proteins (Fig. 2) include a putative polyribonucleotide nucleotidyltransferase (AT5G14580) that harbors three domains, namely an exoribonuclease domain, a K homolog (KH) domain and a S1 domain and a small nuclear ribonucleoprotein Prp4p-related protein (AT2G41500) that contains two domains, the splicing factor motif (SF) and WD40 domain, representing both classical and non-classical RBDs. Some of the candidate proteins contain only one domain but in multiple copies e.g. the pentatricopeptide repeat (PPR) superfamily protein (AT1G18485) that harbors 18 PPR repeats. Diversity of domain organization as shown in the set of selected proteins (Fig. 2) indicates that RBPs come in a wide range of domain organization and may bind to more than one RNA molecule depending on their domain architecture^7^. The presence of multiple RBD and/or copies of the same domain provides the basis for the large structural repertoire that can conceivably expand protein function depending on developmental and physiological conditions^8^. In addition, many RBP candidates, though they share domains with mammalian RBPs, seem to be unique to plants or photosynthetic organisms and may serve specific chloroplast functions.

### The RB-proteome shows enrichments in Gene Ontology (GO) categories

**Figure 3:**
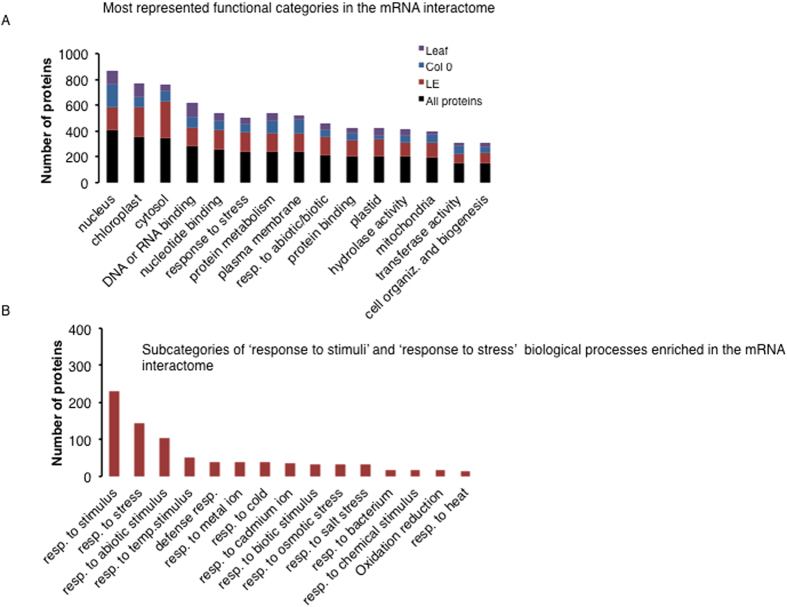
Gene ontology enriched terms in the mRNA interactome proteins. (**A**) Number of proteins in each biological process category. In all categories the mRNA interactome proteins were analyzed (maroon bars), and proteins only unique to Col 0 (blue bars), Ler-0 (green bars) and leaf (purple bars) were also analyzed and their distribution compared. (**B**) Number of proteins in the enriched biological process ‘response to stimuli’ and ‘response to stress’ were plotted and their subcategories included. These biological processes were the most represented categories.

GO analysis using TAIR (https://www.arabidopsis.org/) and AGRIGO (http://bioinfo.cau.edu.cn/agriGO/) facilitated classification of the identified proteins. A molecular function analysis looking at the RB-proteome in terms of functional categories reveals that over 43% of the proteins are annotated as involved in ‘binding’ with about 50% of the proteins in this category involved in ‘DNA or RNA-binding’ (Supplementary Tables S4 and S5). The most represented and enriched (p value < 0.05) subcategories of ‘RNA-binding’ include ‘poly(U) RNA-binding’ (12 proteins), ‘single-stranded RNA-binding’ (12 proteins) and ‘mRNA-binding’ (7 proteins) (Supplementary Table S4). Other functional categories enriched include ‘catalytic activity’ with about 40% of the RBPs. The most represented subcategories in the ‘catalytic activity’ category are ‘hydrolase activity’, representing 14% in the RB-proteome and ‘transferase activity’ representing 13% in the RB-proteome (Fig. 3A, Supplementary Tables S4 and S5). The most represented biological processes include ‘response to stress’ and ‘protein metabolism’ (Fig. 3A). In the former category different stress subcategories were enriched including ‘response to cold’, ‘response to salt’, ‘response to osmotic stress’ and ‘response to heat’ and this does suggest that RBPs may have important functions in responses to abiotic stress (Fig. 3B), a hypothesis that will require further testing.

### Pathway analysis using KEGG

**Figure 4:**
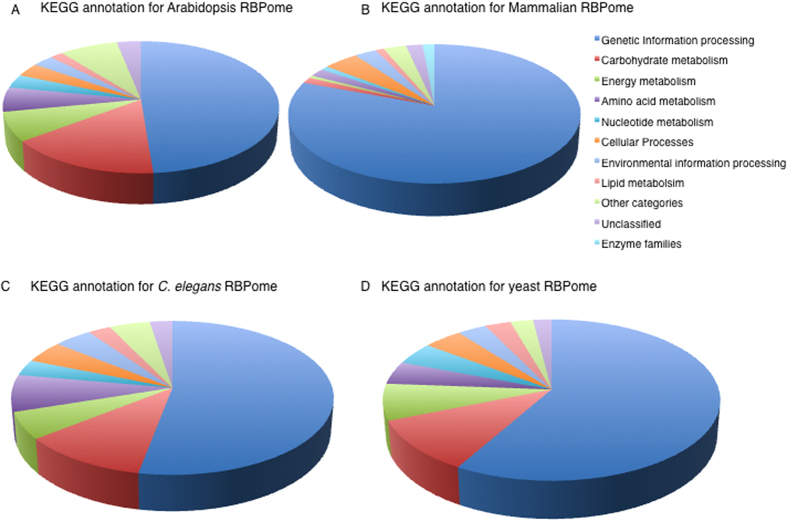
Kyoto encyclopedia of genes and genomes (KEGG) pathway analysis. (**A**) KEGG annotation for Arabidopsis RBP representing 58% of identified proteins that were successfully assigned to pathways. (**B**) KEGG annotation for mammalian RBP representing 78% of identified proteins that were successfully assigned to pathways. (**C**) KEGG annotation for *C. elegans* RBP representing 85% of the identified proteins that could be assigned to pathways. (**D**) KEGG annotation for yeast RBP representing 79% of the identified proteins that were assigned to pathways. The mapped set of proteins include intermediary metabolism pathway and the analysis was performed using KEGG mapper (http://www.kegg.jp/).

To learn if the identified proteins are part of particular metabolic pathways, we performed a metabolic pathway analysis using Kyoto encyclopedia of genes and genomes (KEGG) database. Here, 665 proteins (~55% of the RB-proteome) were assigned to pathways and the ‘genetic information processing’, ‘carbohydrate metabolism’ and ‘energy metabolism’ were the most represented (Fig. 4, Table 1). In the carbohydrate metabolism category that represents one of the intermediary metabolism classes, glycolysis/gluconeogenesis contained the most number of proteins (18 proteins) followed by glyoxylate and dicarboxylate metabolism (15 proteins). The candidate RNA-binding enzymes that belong to the intermediary metabolism do not cluster into a single pathway (Supplementary Table S5). The existence of a post-transcriptional regulatory network interconnecting RBPs, some of which have enzymatic activities, with roles in the intermediary metabolism can be considered moonlighting as previously suggested^40^. The emerging interconnections between *R*NA, *e*nzyme and *m*etabolite (REM) networks may direct post-transcriptional gene regulation. Thus, the dual functional role of proteins acting as enzymes and RBPs can be tuned by metabolites and/or cofactors^40^ and this may be true for plants as well. In this study, proteins with a functional role in intermediary metabolism were identified and examples of the pathways in which they occur include glycolysis, citric acid cycle, pyruvate metabolism and pentose phosphate pathway. It is of interest that some of the enzymes identified in this study such as aconitase (AT4G35830) and enolase (AT2G36530) have been observed to have dual functions, as enzymes and RBPs in other organisms. Aconitase is an iron-sulphur protein and an enzyme of the citric acid cycle that catalyzes the conversion of citrate to isocitrate through a cis-aconitate intermediate under iron-rich conditions^41^. Aconitase has long been known to be a bifunctional enzyme/RBP with well-characterised physiological roles^42^,^43^. In iron-deficient mammalian cells, the iron-sulfur cluster of aconitase is lost and aconitase was observed to bind iron-responsive RNA elements and coordinately regulating the stability of mRNAs encoding functions in iron homeostasis^44^. In *Escherichia coli*, aconitase B has been shown to be a moonlighting protein that can switch to its apo-aconitase form that binds to its own mRNA 3′UTR and stabilising it when intracellular levels of iron are low^45^,^46^. In addition to stabilizing its own mRNA, it also prevents its mRNA from small RNA-induced mRNA cleavage^46^. In bacteria, small RNAs are essential regulators of cellular functions by controlling gene expression in response to environmental changes. The *Bacillus subtilis* aconitase was reported to bind specifically to sequences resembling eukaryotic mRNA iron response elements and this binding is dependent on the availability of iron^47^. Thus, the inactivation of aconitase function as an enzyme is a pre-condition for its RNA-binding role suggesting mutually exclusive states that are regulated by an iron–sulfur cluster acting as a cofactor^40^. Another enzyme, enolase (ENSG00000074800, from HeLa mRNA interactome), has also been shown to interact with RNA and has been experimentally validated through sequencing of RNAs cross-linked to GFP/YFP-fused enolase 1^35^. Based on these lines of evidence, we predict that their plant counterparts, including the aconitase and enolase, may also have bifunctionality. In the mammalian system, other enzymes of the intermediary metabolism have also been implicated in ‘moonlighting’ as RBPs, however, the *in vivo* evidence remains limited^35^.

### Comparing the RBP-proteomes of *A. thaliana*, mammalia, *C. elegans* and *S. cerevisiae*

**Figure 5:**
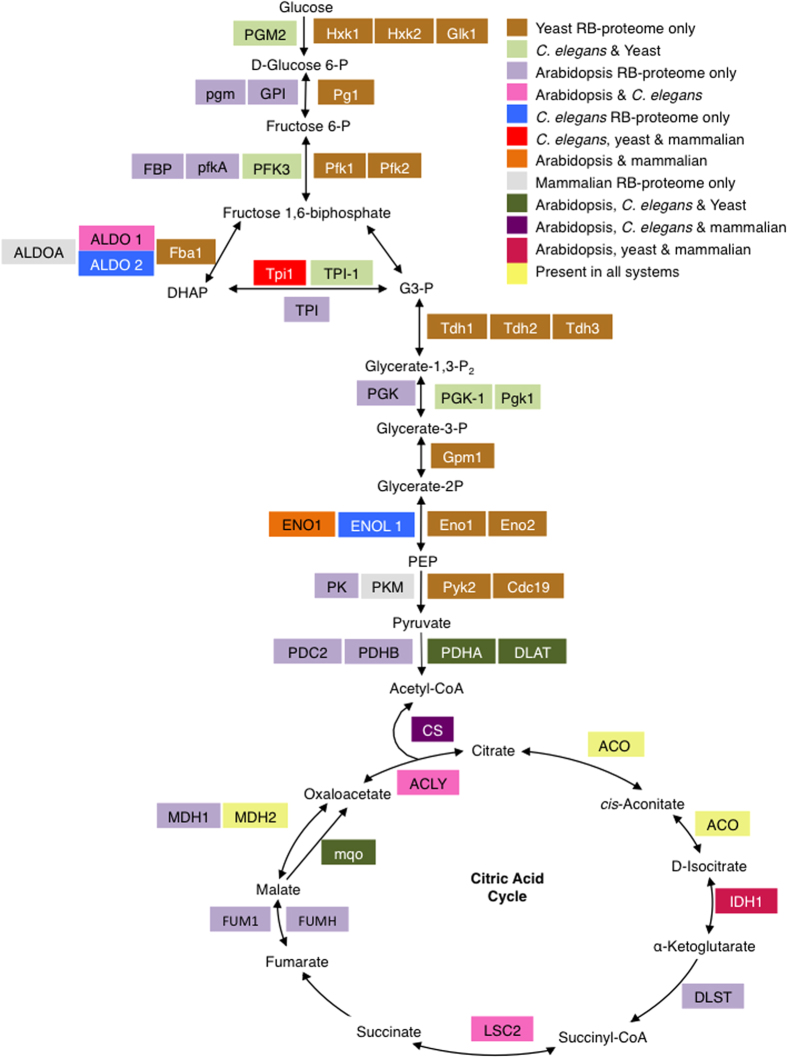
Schematic pathway illustrating the glycolytic (adapted and modified from^50^) and the citric acid pathways. The abbreviations of the enzymes are summarised in Supplementary Table 8. P, phosphate; P_2_, bisP; DHAP, dihydroxyacetone phosphate; PEP, phosphoenolpyruvate; G3-P, glyceraldehyde 3-phosphate.

Cross RB-proteome comparisons were performed using data from (1) mammalian systems comprising mouse embryonic stem cells^30^, proliferating human HeLa cells^35^, human embryonic kidney cell line (HEK 293 cells)^48^, human hepatocytic HuH7 cells^49^, and (2) *C. elegans*^50^, (3) yeast^49^,^50^,^51^ and Arabidopsis RB-proteome reported in this study (Supplementary Table S6). Interrogating the Prosite domain database revealed that the combined dataset from the mammalian samples, *C. elegans*, yeast and Arabidopsis RB-proteomes contains both classical and non-classical RBDs. However, the number of proteins containing classical RBDs are generally greater in mammalian than in Arabidopsis, yeast and *C. elegans* RB-proteomes (Supplementary Figure S2). For example, the RRM domain occurs in the mammalian RB-proteome in at least twice more proteins than in Arabidopsis, four times more than in yeast and 52 times more than in *C. elegans*. The opposite is true for non-classical RBD (Supplementary Figure S2). The presence of the same type of classical RBDs in almost all systems may indicate conserved structure and functional roles of RBD across species. From the current datasets, it could be inferred that some canonical RBDs such as the cold shock domain (CSD) and YTH (YT521-B homology) are not present in *C. elegans* and yeast isolated RB-proteomes and the PAZ and PIWI domains are not present in the *C. elegans*, but are present in mammalian cells and plants. In the non-classical domains, the PPR, a class of relatively well-characterized domains that is prevalent in nature^7^,^52^, was not detected in neither *C. elegans* nor yeast. A global perspective of all possible RBDs based on a recent study^53^ is also summarized here (see Supplementary Table S7). A further comparative analysis was performed on metabolic pathways using KEGG. Only 665 proteins (representing about 55%) of the Arabidopsis RB-proteome could be mapped onto KEGG pathways, while 1017 proteins (representing about 75%) of the mammalian, 848 proteins (representing about 80%) of the yeast and 387 (representing about 85% of the *C. elegans* RB-proteome were mapped. In all systems, the most represented metabolic pathway is the ‘genetic information processing’ pathway that is dominated by proteins associated with the spliceosome, ribosomes and mRNA biogenesis (Fig. 4). Proteins in these categories, particularly those detected in Arabidopsis, were also detected in mammalia, yeast or *C. elegans.* For example, in the Arabidopsis RB-proteome 22 of the 24 proteins assigned to the spliceosome, 49 of the 56 proteins assigned to the translation pathway and 12 of the 14 proteins assigned to ribosome biogenesis have been identified previously in mammalia, yeast or *C. elegans* RB-proteomes^30^,^31^,^48^,^49^,^50^ (Supplementary Table S8). Although ‘genetic information processing’ is the most prevalent category in all systems, it represents about 80% of the proteins of the mammalian RB-proteome mapped, while in yeast and *C. elegans*, it represents only about 55% and in Arabidopsis about 50%. The presence of intermediary metabolism pathways including ‘carbohydrate metabolism’ in the Arabidopsis RB-proteome mapped onto KEGG represents about 15%, 10% in the yeast and *C. elegans* and only just over 1% in the mammalian RB-proteome. It is conceivable that this major difference points to differences in the plant, fungal and animal metabolism and/or may reflect the specific biological and experimental conditions. Among the proteins involved in ‘carbohydrate metabolism’ particularly in the major pathways such as glycolysis, citric acid cycle and pyruvate metabolism, 25 proteins were detected in the RB-proteome of mammalia, yeast or *C. elegans* and are common to the Arabidopsis RB-proteome (Table 2, Supplementary Table S9). Interestingly, of the KEGG assigned RB-proteome, 11 of the 13 Arabidopsis citric acid cycle proteins (Fig. 5), 16 of the 18 glycolysis proteins (Fig. 5) and 11 of the 12 pentose phosphate pathway proteins had previously been detected in the RB-proteome of other organisms^30^,^31^,^48^,^49^,^50^ (Table 2, Supplementary Table S9). This set of proteins includes enolase, a protein involved in glycolysis, that, as described earlier, was experimentally proven to bind RNA molecules in HeLa cells^35^. As expected, proteins involved in the photosynthesis pathway and the photosynthesis antenna were only observed in plant tissue. Overall, a comparison of Arabidopsis, mammalian, *C. elegans*, and yeast RBPs reveals a common set of proteins with a role in intermediate metabolism, as well as distinct differences suggesting that RBPs are both species and tissue specific and almost certainly responsive to stimuli.

### Validation of novel candidate RBPs

**Figure 6:**
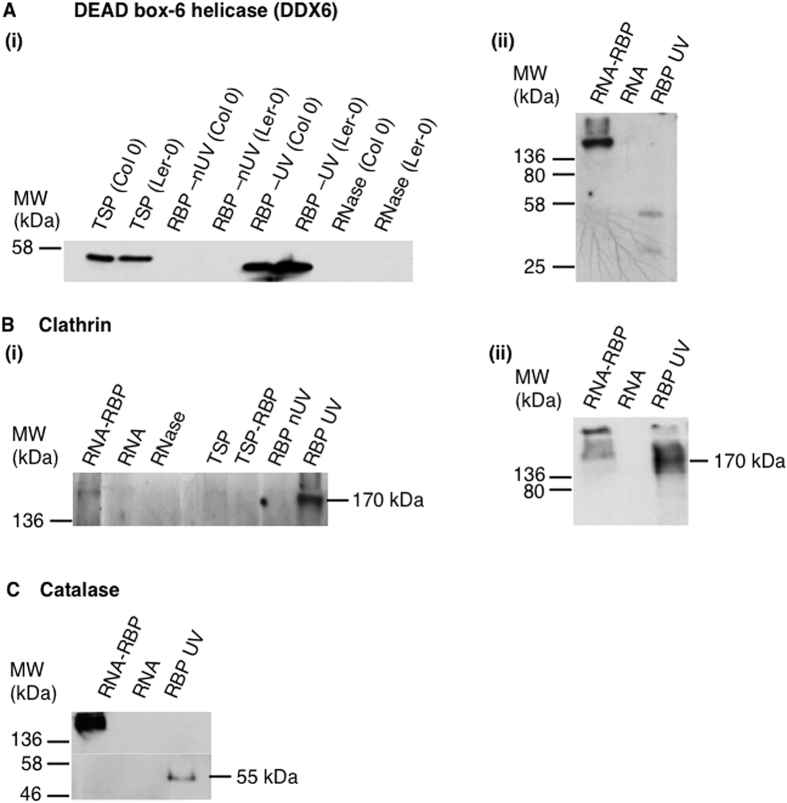
Validation of RNA-binding proteins with a western blot analysis. (**A**) Western blots showing (i) total soluble protein (TSP) from Arabidopsis Col 0 ecotype, TSP from Landsberg erecta (Ler-0), RBP non UV (nUV)-crosslinked Col 0, RBP nUV-crosslinked Ler-0, RBP UV-crosslinked Col 0, RBP UV-crosslinked Ler-0 and RNAse treated samples from Col 0 and Ler-0 and (ii) RNA-RNA-binding protein (RNA-RBP) complex, RNA extract, RBP UV-crosslinked samples probed with antibodies against the DEAD box-6 helicase (DDX6). (**B**) Western blot showing (i) RNA-RBP complex, RNA extract, RNAse treated sample, TSP, TSP sample after extracting RBP (TSP-RBP), RBP nUV-crosslinked and RBP UV-crosslinked samples and (ii) RNA-RBP complex, RNA extract and RBP UV-crosslinked samples probed with antibodies against the clathrin heavy chain 1 (AT3G11130). (**B**) (i) and (ii) are two independent runs performed to check reproducibility of the validation experiment. (**C**) Western blot showing RNA-RBP complex, RNA extract and RBP UV-crosslinked samples probed with antibodies against catalase 3 (AT1G20620).

To validate novel and conserved RBPs identified in this study, we used a western blot-based assay. The technique utilizes the power of antibody specificity against target proteins. We UV-crosslinked proteins to mRNAs *in vivo* and performed oligo(dT) capture. The RNA-RBP complex, and isolated RBPs and RNA samples were analyzed by western blotting using antibodies against DEAD box-6 helicase (DDX6) as a positive control, and clathrin heavy chain and catalase, a peroxisomal protein, as novel candidate RBPs for validation. The blots showed distinct positional band shifts between the RNA-RBP complex sample and the RBP sample for all the antibodies tested (Fig. 6). Here, we tested the UV-crosslinked RNA-protein complex to show a band shift from an uncrosslinked protein. The validity of the assay was confirmed by the specific reactivity of the DDX6 antibody that caused a distinct band shift between the RNA-DDX6 complex (band size >136 kDa) and the DDX6 protein (~55 kDa). The DDX6 is a well characterized RNA-binding protein. We detected no bands with the mRNA sample as well as with RNase treated and the RBP nUV-crosslinked samples (Fig. 6A). The novel candidate RBPs, clathrin heavy chain and catalase revealed the same positional band shift between the RNA-RBP complex and the RBP samples (Fig. 6B,C). In the RBP samples, bands corresponding to the predicted molecular weight were obtained for each protein and the RNA-RBP complex consistently had an increased molecular weight compared to the target protein alone.

Two clathrin heavy chains, AT3G11130 and AT3G08530, were identified as novel candidate RBPs. AT3G11130 was the only protein detected in both Col 0 and Ler-0 cell cultured cells, while AT3G08530 was identified only in Ler-0. Since the validation experiment was performed on the Col 0 sample, we targeted AT3G11130. Clathrin is a protein that comprises three heavy chains and three light chains. It is involved in intracellular trafficking and plays a major role in the formation of coated vesicles that selectively sort cargo at the cell membrane, trans-Golgi network and endosomal compartments. The Arabidopsis clathrin heavy chain 1 (AT3G11130) comprises four different domains, namely clathrin heavy chain or VPS 7-fold repeat, clathrin heavy chain linker or propeller domain, armadillo-type fold and tetratricopeptide-like helical domain, none of which have yet been directly associated with mRNA binding. However, the presence of the tetratricopeptide-like helical domain, that comprises superhelical structures that are well suited to binding large molecules including nucleic acids and proteins^54^, does hint to a role of clathrin in mRNA binding. The topology exhibited by the tetratricopeptide-like helical domain has been detected in pentatricopeptide repeats that are mRNA interacting domains found in mRNA processing proteins^54^.

Catalase 3 (AT1G20620), a peroxisomal protein containing only catalase-related and haem-dependent domains, catalyzes the breakdown of hydrogen peroxide into water and oxygen making it a part of the plant antioxidative system. Since catalases are highly expressed enzymes and have a fast turnover rate^55^, mRNA-binding may prove critical for their turn-over and functional regulation much like in the specific enzyme-mRNA interaction observed in the human glycolytic enzymes^50^.

## Conclusion

The interactome capture technique which combines UV-crosslinking and oligo(dT) capture and mass spectrometry has provided a platform to identify RBPs in plants, at the systems level. Although novel RBP candidates have been identified, not all previously annotated RBP were identified in this study, suggesting that some of the RBPs may be stimulus specifically induced and/or tissue- and developmental stage-specific. This conjecture is partly supported by enriched GO categories where RBPs could be clustered into specific biological process and molecular function categories.

In summary, this study provides a platform for systems scale studies aiming at identifying and characterizing novel RBPs and their role in response to stresses such as salinity, drought and cold. Future investigations will include, firstly, further validation of more novel candidate RBPs, secondly, the identification of novel specific RNA-peptide interacting regions in plants as well as the elucidation of the role of posttranslational modifications in the regulating RNA-protein interactions. Thirdly, future work also aim to identify the RNA targets of novel RBPs.

## Methods

### Cell culture and plant growth

Cells derived from roots of *Arabidopsis thaliana*, ecotypes Columbia-0 and Landsberg erecta (Ler-0) were grown in liquid culture as previously described^56^,^57^,^58^,^59^. Three biological replicates of cells were collected. Media were drained using Stericup^®^ filter units (EMD Millipore, Billerica, MA, USA) and cells were rinsed with 1× phosphate buffered saline immediately before UV-crosslinking. For whole plant work, Arabidopsis plants (ecotype Col-0) were grown under controlled conditions: 22 °C, 100 μmol m^−2^ s^−1^, and relative humidity of 70%. Four week-old plants were subjected to UV-crosslinking, as described previously^31^.

### UV-crosslinking and interactome capture

*In vivo* crosslinking and isolation of Arabidopsis RBPs was performed, as detailed described^31^. Briefly, each flask of cells was divided into two, to approximately one gram of cells per biological replicate. Cells were placed onto Petri dishes and one of each pair was used for UV-crosslinking (UV) and the other for the control (no UV-crosslinking (nUV)). Cells for UV-crosslinking were irradiated with 0.15 J/cm^2^ UV light at 254 nm for ~90 s, lysed in lysis buffer (20 mM Tris (pH 7.5), 500 mM LiCl, 0.5% (w/v) lithium dodecyl sulfate, 1 mM EDTA, 5 mM DTT) using a PowerGen 125 grinder (ThermoFisher Scientific, Rockford, IL, USA), vortexed for 1 min and incubated on ice for 10 min. Debris were pelleted by using an Allegra^®^ X-22R centrifuge (Beckman Coulter Corp., Brea, CA, USA) at 400 × *g* for 10 min at 4 °C and the supernatant was carefully collected. Oligo(dT)_25_ magnetic beads (NEB, S1419S) were used to capture poly(A)^+^ RNAs, undergo several washes prior to elution of mRNA-protein complexes, as described elsewhere^31^. To assess the quality of the mRNA-protein crosslinked complex pull down, an additional control was performed by treating the sample with RNase T1/A mix (ThermoFisher Scientific) and the reaction was performed according to the manufacturer’s recommendation. For RBP analysis samples were treated with RNase A/T1 mix to release them from the captured RNA molecules. Proteins were then analyzed by western blotting using antibodies against polypyrimidine tract-binding protein 1, β-actin (Sigma Aldrich, St Louis, MO, USA) and Histone 3 (Abcam, Cambridge, UK) following the manufacturers’ recommendations.

### Protein digestion and mass spectrometry

Protein samples were reduced, alkylated, buffer exchanged and digested, as described previously^30^,^31^ with the following modifications. Samples were adjusted to 5 mM DTT, incubated for 1 h at 56 °C and then 200 μl of 8 M urea in 0.1 M Tris-HCl (pH 8.5) was added and the sample was concentrated using Amicon Ultra Centrifugal Filters (0.5 mL, 3-kDa cut-off, Millipore, Billerica, MA, USA) at 16,000 × *g* for 30 min at 20 °C. Then 100 μl of 50 mM iodoacetamide was added, the samples were mixed at 600 r.p.m for 1 min using a benchtop mixer (ThermoFisher Scientific) and incubated for 30 min at room temperature in the dark and then concentrated. Buffer exchange was performed by adding 100 μl of 8 M urea in 0.1 M Tris-HCl (pH 8.0) and concentrated, then 100 μL 50 mM NH_4_HCO_3_ was added and the sample was concentrated again in three successive rounds. A 1 μg of trypsin in 120 μl of 50 mM NH_4_HCO_3_ was added and the filter units were incubated at room temperature overnight. The derived peptides were collected by centrifugation of the filter units. The filters were washed with 50 μL of 0.5 M NaCl and centrifuged again. The collected peptides were acidified with TFA and desalted using Sep-Pak cartridges (Vac 1cc, 50 mg, tC18) (Waters, Milford, MA, USA) as described elsewhere^60^. Samples were dried and resuspended in 20 μl of 5% (v/v) acetonitrile and 0.1% (v/v) formic acid and analyzed with LTQ-Orbitrap Velos MS (Thermo-Scientific) coupled with a nanoelectrospray ion source (Proxeon Biosystems, Odense, Denmark) as described elsewhere^60^.

Discover v1.2.0.208 (Thermo Scientific) and submitted to a local MASCOT (Matrix Science, London, UK) server and searched against *Arabidopsis thaliana* in the Arabidopsis information resource (TAIR; release 10) with precursor mass tolerance of 20 ppm, a fragment ion mass tolerance of ±0.5 Da and strict trypsin specificity allowing up to one missed cleavage, peptide charges of +1, +2 and +3, carbamidomethyl modification on cysteine residues as fixed modification. Further stringency was applied to the protein identification by considering only proteins with a molecular weight search (MOWSE) score ≥ 32 (95% confidence limit). Data was further analyzed and validated with Scaffold v4.0.4 (Proteome Software, Portland, OR, USA) allowing for 0.1% FDR^58^ calculated using the decoy database.

### UV-crosslink enrichment

Proteins that were detected in both the UV-crosslinked samples and the control (non-UV crosslinked samples) were quantitatively analyzed to assess UV-crosslinking enrichment. Spectral counts were normalized, and a fold change and *p*-value (using Student’s T-test) were calculated. UV-crosslinking enrichment was determined by employing a fold increase of ≥1.5 and a *p*-value of ≤0.05.

### Systems data analysis

Classical and non-classical RNA-binding domains were detected from all proteins using prosite (http://prosite.expasy.org/scanprosite/; October 2015). Gene ontology (GO) enrichments were performed using AGRIGO (http://bioinfo.cau.edu.cn/agriGO/) and tair (https://www.arabidopsis.org/; October 2015). The pathway analysis was done with the KEGG mapper (http://www.kegg.jp/kegg/tool/annotate_sequence.html; October 2015).

### Assigning novel *A. thaliana* RBP-proteomes

In order to assign an RBP as novel and specific to Arabidopsis, a cross RB-proteome comparison was performed using data from (1) the mammalian systems comprising mouse embryonic stem cells^30^, proliferating human HeLa cells^35^, human embryonic kidney cell line (HEK 293 cells)^48^, human hepatocytic HuH7 cells^49^, (2) *C. elegans*^50^, (3) yeast^49^,^50^,^51^ and the Arabidopsis RB-proteome reported in this study. Following this analysis, proteins were assigned as novel candidate RBP when (1) the protein had no known RBD, as predicted using the Prosite domain database, (2) was not assigned to any molecular function associated with RNA binding, RNA processing or modification and (3) had no common KEGG accession with mammalia, *C. elegans* or *C. cerevisae* as assigned using the KEGG mapper.

### Validation of novel candidate RBPs

In order to validate some of the novel candidate RBPs identified in this study, a western blot-based assay was performed. *In vivo* UV-crosslinking and isolation of Arabidopsis Col 0 RNA-RBPs was performed, as described earlier. Following the capture poly(A)^+^ RNAs using Oligo(dT)_25_ magnetic beads, the eluted sample was split into three fractions. One fraction was directly concentrated using a freeze drier to concentrate RNA-RBP complexes, the second fraction was used for RBP isolation as described previously^30^,^31^ and the third fraction was used for RNA isolation as described previously^30^,^31^. The samples were fractionated on a native gel and then analyzed by western blotting using antibodies against DEAD box-6 helicase (DDX6; Universal biologicals, Cambridge, UK) as a control, and clathrin heavy chain and catalase, a peroxisomal protein (Agrisera, Sweden), as novel RBP for validation following the manufacturers’ recommendations.

## Additional Information

**How to cite this article**: Marondedze, C. *et al*. The RNA-binding protein repertoire of *Arabidopsis thaliana* *Sci. Rep.* **6**, 29766; doi: 10.1038/srep29766 (2016).

## Supplementary Material

Supplementary Table S1

Supplementary Table S2

Supplementary Table S3

Supplementary Table S4

Supplementary Table S5

Supplementary Table S6

Supplementary Table S7

Supplementary Table S8

Supplementary Table S9

Supplementary Information

## Figures and Tables

**Table 1:** Most represented KEGG pathways in the mRNA interactome (containing ≥10 proteins).

KEGG accession#	KEGG term	#proteins
Carbohydrate metabolism		
00010	Glycolysis/Gluconeogenesis	18
00020	Citrate cycle (TCA)	13
00030	Pentose phosphate pathway	12
00500	Starch and sucrose metabolism	12
00520	Amino sugar and nucleotide sugar metabolism	12
00620	Pyruvate metabolism	14
00630	Glyoxylate and dicarboxylate metabolism	15
Energy metabolism		
00190	Oxidative phosphorylation	17
00195	Photosynthesis	14
00710	Carbon fixation in photosynthetic organisms	15
Nucleotide metabolism		
00230	Purine metabolism	20
00240	Pyrimidine metabolism	14
00260	Glycine, serine and threonine metabolism	16
00270	Cysteine and methionine metabolism	13
Transcription		
03040	Spliceosome	24
Translation		
03008	Ribosome biogenesis in eukaryotes	14
03010	Ribosome	56
03013	RNA transport	21
03015	mRNA surveillance pathway	10
00970	Aminoacyl-tRNA biosynthesis	12
Folding, sorting and degradation		
04141	Protein processing in endoplasmic reticulum	10
03050	Proteasome	12
03018	RNA degradation	12

**Table 2:** Representative RBPs in the intermediary metabolism that are detected in Arabidopsis, animal systems and yeast.

KEGG pathway	KEGG ID	Arabidopsis	Mammal	*C. elegans*	Yeast	Protein description
00010 Glycolysis/Gluconeogenesis						
	K14085	AT1G54100		P46562		Aldehyde dehydrogenase 7B4
	K01835	AT1G70730			P33401	Phosphoglucomutase
	K01810	AT5G42740			P12709	Sugar isomerase
	K01803	AT2G21170	P17751	Q10657	P00942	Triosephosphate isomerase
	K01792	AT5G57330			Q03161	Galactose mutarotase-like
	K01689	AT1G74030	P06733	Q27527	P00924	Enolase 1
	K01623	AT2G21330	J3KPS3	P54216		Fructose-bisphosphate aldolase 1
	K01568	AT5G54960			P06169	Pyruvate decarboxylase-2
	K00927	AT1G79550		P91427	P00560	Phosphoglycerate kinase
	K00873	AT5G63680	P14618		P00549	Pyruvate kinase
	K00850	AT4G26270			P16861	Phosphofructokinase 3
	K00627	AT3G13930		Q19749	P12695	Dihydrolipoamide acetyltransferase
	K00382	AT1G48030	P09622		P09624	Mitochondria lipoamide dehydrogenase 1
	K00162	AT5G50850		O44451	P32473	Transketolase family protein
	K00161	AT1G01090		P52899	P16387	Pyruvate dehydrogenase E1 alpha
	K00121	AT5G43940		Q17335		GroES-like zinc-binding dehydrogenase
00020 Citrate cycle (TCA cycle)						
	K01900	AT2G20420		P53588		ATP citrate lyase
	K01681	AT2G05710	P21399	Q23500	P19414	Aconitase 3
	K01679	AT2G47510		O17214	P08417	Fumarase 1
	K01648	AT5G49460		P53585		ATP citrate lyase subunit B2
	K01647	AT2G44350	O75390	P34575		Citrate synthase
	K00627	AT3G13930		Q19749	P12695	Dihydrolipoamide acetyltransferase
	K00382	AT1G48030	P09622		P09624	Mitochondria lipoamide dehydrogenase 1
	K00162	AT5G50850		O44451	P32473	Transketolase family protein
	K00161	AT1G01090		P52899	P16387	Pyruvate dehydrogenase E1 alpha
	K00031	AT1G54340	O75874		P21954	Isocitrate dehydrogenase
	K00026	AT3G15020	P40926	O02640	P17505	Lactate/malate dehydrogenase
00620 Pyruvate metabolism						
	K14085	AT1G54100		P46562		Aldehyde dehydrogenase 7B4
	K11262	AT1G36160			Q00955	Acetyl-CoA carboxylase 1
	K01679	AT2G47510		O17214	P08417	Fumarase 1
	K01649	AT1G18500			P06208	Methylthioalkylmalate synthase-like 4
	K00873	AT5G63680	P14618		P00549	Pyruvate kinase
	K00627	AT3G13930		Q19749	P12695	Dihydrolipoamide acetyltransferase
	K00382	AT1G48030	P09622		P09624	Mitochondria lipoamide dehydrogenase 1
	K00162	AT5G50850		O44451	P32473	Transketolase family protein
	K00161	AT1G01090		P52899	P16387	Pyruvate dehydrogenase E1 alpha
	K00026	AT3G15020	P40926	O02640	P17505	Lactate/malate dehydrogenase
