# Supplementary material for: The RNA-binding protein repertoire of Arabidopsis thaliana
Source: Sci Rep. 2016 Jul 11;6:29766. doi: 10.1038/srep29766 (PMC4942612; doi:10.1038/srep29766)
Supplement: Supplementary Information [file srep29766-s10.doc]

**The RNA-binding protein repertoire of *Arabidopsis thaliana***

Claudius Marondedze, Ludivine Thomas, Natalia L. Serrano, Kathryn S. Lilley, Chris Gehring

**Supplementary Information**

**Supporting Tables**

**Table S1.** Proteins identified from the Arabidopsis Col 0 and Ler-0 cell cultures, and Leaves from the Arabidopsis ecotype Col 0 plants.

**Table S2.** Set of proteins enriched by UV-crosslinking.

**Table S3.** Set of previously unknown Arabidopsis domains that interact with RNA.

**Table S4.** Gene ontology enrichment in the Arabidopsis RB-proteome.

**Table S5.** Functional categorization and pathway analysis of Arabidopsis RNA-binding proteins.

**Table S6.** Arabidopsis, mammalian, *C. elegans* and yeast RNA-binding proteomes used for comparative analysis.

**Table S7.** List of protein families (Pfam) domains associated with RB-proteomes. This modified list is based on a recent classification system ^53^ and the data used in this table is based on the data from this current study as well as the experimental data from mammalian system ^30,31,35,48,49^, *C. elegans^50^* and yeast ^49-51^.

**Table S8.** KEGG pathways assigned to the Arabidopsis, mammalian, *C. elegans* and yeast RB-proteome.

**Table S9.** Common proteins between Arabidopsis, mammalian samples, *C. elegans* and yeast RNA interactome involved in intermediary metabolism.

**The RNA-binding protein repertoire of *Arabidopsis thaliana***

Claudius Marondedze, Ludivine Thomas, Natalia L. Serrano, Kathryn S. Lilley, Chris Gehring

**Supplementary Information**

**Supporting Figures**

**Supplementary Figure S1.** Western blot analysis and Venn diagram showing the RNA-binding proteomes in cell cultures and leaf samples. **A.** Western blot showing protein extracts, total soluble protein (TSP), UV-crosslinked (UV), non UV-crosslinked (nUV) and RNase treated extract analyzed against antibodies for polypyrimidine tract binding protein 1 (PTBP1), β-actin, histone H3. **B.** Total number of enriched in UV cross-linked mRNA interactome proteins in the cell suspension cultures of Arabidopsis Col 0 and Landsberg erecta, and leaf samples. These proteins were detected in three biological replicates. **C.** Total number of enriched proteins (after UV cross-linking) in the cell suspension cultures of Arabidopsis Col 0 and Landsberg erecta, and leaf samples when identification parameters are relaxed. These proteins were detected in three biological replicates.

**The RNA-binding protein repertoire of *Arabidopsis thaliana***

Claudius Marondedze, Ludivine Thomas, Natalia L. Serrano, Kathryn S. Lilley, Chris Gehring

**Supplementary Figure S2.** Comparison of the globular domains in Arabidopsis, mammalian, *C. elegans* and yeast mRNA interactome proteins. **A.** Number of proteins harboring classical RBD in the mRNA interactomes. **B.** Number of proteins harboring non-classical RBD. **C.** Summary of the total number of proteins harboring classical, non-classical and unknown RBD.
